# *N*-[(*E*)-4-Chloro­benzyl­idene]-*N*′-phenyl­benzene-1,4-diamine

**DOI:** 10.1107/S160053681002742X

**Published:** 2010-07-17

**Authors:** Nor Zakiah Nor Hashim, Karimah Kassim, Bohari M. Yamin

**Affiliations:** a Department of Chemistry, Faculty of Applied Sciences, Universiti Teknologi MARA, 40450 Shah Alam, Selangor, Malaysia; b School of Chemical Sciences and Food Technology, Universiti Kebangsaan Malaysia, UKM 43600 Bangi Selangor, Malaysia

## Abstract

The title compound, C_19_H_15_ClN_2_, adopts an *E* configuration with respect to the position of the chloro­benzene and diphenyl­amine groups on the C=N azomethine bond. The mol­ecule is not planar, the central six-membered ring making angles of 12.26 (10) and 44.18 (11)° with the 4-chloro­phenyl and phenyl rings, respectively. In the crystal structure, weak C—H⋯π inter­actions contribute to the stabilization of the packing.

## Related literature

For related structures, see: Ojala *et al.* (2007 ▶); Fun *et al.* (2008 ▶). For standard bond lengths, see: Allen *et al.* (1987 ▶). For the biological activity of Schiff bases, see: Küstü *et al.* (2007 ▶) and for their pharmaceutical properties and applications as corrosion inhibitors, see: Singh & Dhakarey (2009 ▶).
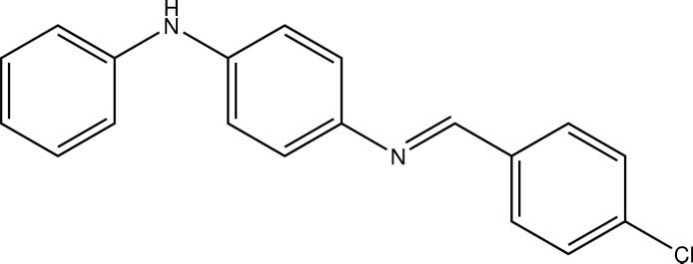


## Experimental

### 

#### Crystal data

C_19_H_15_ClN_2_
*M* *_r_* = 306.78
Monoclinic, 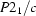

*a* = 10.3353 (15) Å
*b* = 17.045 (3) Å
*c* = 8.7893 (13) Å
β = 97.384 (3)°
*V* = 1535.5 (4) Å^3^
*Z* = 4
Mo *K*α radiation
μ = 0.25 mm^−1^
*T* = 298 K
0.50 × 0.39 × 0.12 mm

#### Data collection

Bruker SMART APEX CCD area-detector diffractometer
Absorption correction: multi-scan (*SADABS*; Bruker, 2000 ▶) *T*_min_ = 0.886, *T*_max_ = 0.971
8939 measured reflections
2860 independent reflections
2076 reflections with *I* > 2/s(*I*)
*R*_int_ = 0.038

#### Refinement

*R*[*F* ^2^ > 2σ(*F* ^2^)] = 0.054
*wR*(*F* ^2^) = 0.127
*S* = 1.05
2860 reflections
199 parameters
H-atom parameters constrained
Δρ_max_ = 0.22 e Å^−3^
Δρ_min_ = −0.27 e Å^−3^

### 

Data collection: *SMART* (Bruker, 2000 ▶); cell refinement: *SAINT* (Bruker, 2000 ▶); data reduction: *SAINT*; program(s) used to solve structure: *SHELXS97* (Sheldrick, 2008 ▶); program(s) used to refine structure: *SHELXL97* (Sheldrick, 2008 ▶); molecular graphics: *SHELXTL* (Sheldrick, 2008 ▶); software used to prepare material for publication: *SHELXTL*, *PARST* (Nardelli, 1995 ▶) and *PLATON* (Spek, 2009 ▶).

## Supplementary Material

Crystal structure: contains datablocks global, I. DOI: 10.1107/S160053681002742X/zq2049sup1.cif

Structure factors: contains datablocks I. DOI: 10.1107/S160053681002742X/zq2049Isup2.hkl

Additional supplementary materials:  crystallographic information; 3D view; checkCIF report

## Figures and Tables

**Table 1:** Hydrogen-bond geometry (Å, °) *Cg*1 and *Cg*3 are the centroids of the C1–C6 and C14–C19 rings, respectively.

*D*—H⋯*A*	*D*—H	H⋯*A*	*D*⋯*A*	*D*—H⋯*A*
C1—H1*B*⋯*Cg*3^i^	0.93	2.95	3.661 (2)	135
C16—H16*A*⋯*Cg*1^ii^	0.93	2.90	3.624 (2)	136

Symmetry codes: (i) 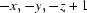; (ii) 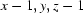.

**Fig. 1.**
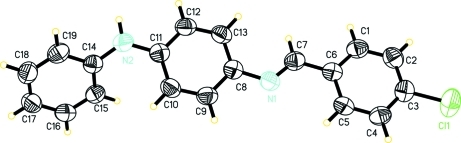
The molecular structure of (I) with displacement ellipsoids drawn at the 50% probability level.

## supplementary crystallographic information

### Comment

The continuing study on Schiff bases are driven not only because of their application as ligands but also because of their biological (Singh & Dhakarey, 2009) and pharmaceutical properties and as corrosion inhibitors (Küstü *et al.*, 2007).

The title compound, C_19_H_15_N_2_Cl (I), is a Schiff base having chlorobenzylidene and phenyl groups attached at the terminal nitrogen atoms of the 1,4-diaminobenzene group (Fig.1). The whole molecule is not planar. Each benzene ring is planar with a maximum deviation of 0.011 (2) Å for the C6 atom from the (C1—C6) ring. The middle (C8—C13) ring makes angles of 12.26 (10)° and 44.18 (11)° with the (C1—C6) and (C14—C19) rings, respectively. The dihedral angle between (C1—C6) and (C14—C19) rings is 56.00 (11)°. The *E* conformation about the C7=N1 double bond is also observed in *N*,*N*' -bis(2-methoxybenzylidene)-*p*-phenylenediamine (II) with an angle of 12.10 (15)° between the mean planes of the benzene rings (Ojala *et al.*, 2007). The bond lengths and angles are in normal ranges (Allen *et al.*, 1987) and comparable to those in (II) and 2-{(4-(phenyldiazenyl)phenl]imino-methyl}phenol (Fun *et al.*, 2008).

In the crystal structure, the molecule is stablized by C—H..π interactions, C1—H1B ···*Cg*3 (C14—C19) and C16—H16A···*Cg*1 (C1—C6) with H···*Cg* distances of 2.95 and 2.90 Å, and C—H···*Cg* angles of 135 and 136°, respectively.

### Experimental

4-Chlorobenzaldehyde (0.7029 g, 0.005 mol) in 15 ml of ethanol and *N*-phenyl-1,4-phenylenediamine (0.9212 g, 0.005 mol) in 10 ml of ethanol were mixed in a round bottom flask. The mixture was stirred for 30 minutes at about 30 °C. The mixture was left to cool down in an ice bath. A green solid was collected and washed with cold ethanol. Green crystals were obtained by recrystallization from toluene (yield 72%; melting point: 408–411 K; CHNS: C, 74.38; H, 4.93; N, 9.13. Found: C, 74.09; H, 4.91; N, 9.08. IR (cm^-1^): C=N, 1592; N—H, 3408; C—Cl, 749.

### Refinement

The H atoms were positioned geometrically with C—H = 0.93 and N—H = 0.86 Å and constrained to ride on their parent atoms with *U*_iso_(H)= 1.2 *x U*_eq_ (C or N).

### Crystal data

**Table:**

C_19_H_15_ClN_2_	*F*(000) = 640
*M**_r_* = 306.78	*D*_x_ = 1.327 Mg m^−^^3^
Monoclinic, *P*2_1_/*c*	Mo *K*α radiation, λ = 0.71073 Å
Hall symbol: -P 2ybc	Cell parameters from 1956 reflections
*a* = 10.3353 (15) Å	θ = 1.9–25.5°
*b* = 17.045 (3) Å	µ = 0.25 mm^−^^1^
*c* = 8.7893 (13) Å	*T* = 298 K
β = 97.384 (3)°	Block, colourless
*V* = 1535.5 (4) Å^3^	0.50 × 0.39 × 0.12 mm
*Z* = 4	

### Data collection

**Table:**

Bruker SMART APEX CCD area-detector diffractometer	2860 independent reflections
Radiation source: fine-focus sealed tube	2076 reflections with *I* > 2/s(*I*)
graphite	*R*_int_ = 0.038
Detector resolution: 83.66 pixels mm^-1^	θ_max_ = 25.5°, θ_min_ = 1.9°
ω scan	*h* = −11→12
Absorption correction: multi-scan (*SADABS*; Bruker, 2000)	*k* = −14→20
*T*_min_ = 0.886, *T*_max_ = 0.971	*l* = −10→9
8939 measured reflections	

### Refinement

**Table:**

Refinement on *F*^2^	Primary atom site location: structure-invariant direct methods
Least-squares matrix: full	Secondary atom site location: difference Fourier map
*R*[*F*^2^ > 2σ(*F*^2^)] = 0.054	Hydrogen site location: inferred from neighbouring sites
*wR*(*F*^2^) = 0.127	H-atom parameters constrained
*S* = 1.05	*w* = 1/[σ^2^(*F*_o_^2^) + (0.0491*P*)^2^ + 0.388*P*] where *P* = (*F*_o_^2^ + 2*F*_c_^2^)/3
2860 reflections	(Δ/σ)_max_ = 0.001
199 parameters	Δρ_max_ = 0.22 e Å^−^^3^
0 restraints	Δρ_min_ = −0.27 e Å^−^^3^

### Special details

**Table:**

Geometry. All e.s.d.'s (except the e.s.d. in the dihedral angle between two l.s. planes) are estimated using the full covariance matrix. The cell e.s.d.'s are taken into account individually in the estimation of e.s.d.'s in distances, angles and torsion angles; correlations between e.s.d.'s in cell parameters are only used when they are defined by crystal symmetry. An approximate (isotropic) treatment of cell e.s.d.'s is used for estimating e.s.d.'s involving l.s. planes.
Refinement. Refinement of *F*^2^ against ALL reflections. The weighted *R*-factor *wR* and goodness of fit *S* are based on *F*^2^, conventional *R*-factors *R* are based on *F*, with *F* set to zero for negative *F*^2^. The threshold expression of *F*^2^ > σ(*F*^2^) is used only for calculating *R*-factors(gt) *etc*. and is not relevant to the choice of reflections for refinement. *R*-factors based on *F*^2^ are statistically about twice as large as those based on *F*, and *R*- factors based on ALL data will be even larger.

### Fractional atomic coordinates and isotropic or equivalent isotropic displacement parameters (Å^2^)

**Table:**

	*x*	*y*	*z*	*U*_iso_*/*U*_eq_	
Cl1	0.49291 (8)	0.14092 (4)	1.51538 (8)	0.0878 (3)	
N1	0.19891 (17)	0.12993 (11)	0.7853 (2)	0.0563 (5)	
N2	−0.00657 (18)	0.08239 (12)	0.1689 (2)	0.0649 (6)	
H2A	0.0363	0.0492	0.1212	0.078*	
C1	0.3831 (2)	0.03701 (13)	1.1159 (3)	0.0595 (6)	
H1B	0.3918	−0.0123	1.0730	0.071*	
C2	0.4384 (2)	0.05077 (14)	1.2645 (3)	0.0614 (6)	
H2B	0.4851	0.0116	1.3210	0.074*	
C3	0.4233 (2)	0.12328 (14)	1.3281 (3)	0.0568 (6)	
C4	0.3532 (2)	0.18165 (13)	1.2465 (3)	0.0583 (6)	
H4A	0.3421	0.2301	1.2915	0.070*	
C5	0.2997 (2)	0.16732 (13)	1.0975 (3)	0.0540 (6)	
H5A	0.2526	0.2067	1.0418	0.065*	
C6	0.3150 (2)	0.09492 (13)	1.0290 (2)	0.0502 (5)	
C7	0.2607 (2)	0.07919 (13)	0.8699 (3)	0.0556 (6)	
H7A	0.2723	0.0296	0.8296	0.067*	
C8	0.1486 (2)	0.11346 (13)	0.6310 (2)	0.0506 (5)	
C9	0.0617 (2)	0.16743 (13)	0.5585 (3)	0.0565 (6)	
H9A	0.0388	0.2110	0.6130	0.068*	
C10	0.0079 (2)	0.15853 (13)	0.4074 (3)	0.0574 (6)	
H10A	−0.0497	0.1960	0.3615	0.069*	
C11	0.0396 (2)	0.09396 (13)	0.3239 (3)	0.0514 (5)	
C12	0.1269 (2)	0.03969 (14)	0.3963 (3)	0.0575 (6)	
H12A	0.1493	−0.0040	0.3418	0.069*	
C13	0.1808 (2)	0.04887 (13)	0.5455 (3)	0.0561 (6)	
H13A	0.2395	0.0117	0.5907	0.067*	
C14	−0.1137 (2)	0.11789 (12)	0.0811 (3)	0.0500 (5)	
C15	−0.2251 (2)	0.13990 (12)	0.1426 (3)	0.0532 (6)	
H15A	−0.2297	0.1331	0.2468	0.064*	
C16	−0.3291 (2)	0.17186 (13)	0.0496 (3)	0.0581 (6)	
H16A	−0.4032	0.1871	0.0920	0.070*	
C17	−0.3251 (2)	0.18156 (14)	−0.1047 (3)	0.0649 (6)	
H17A	−0.3957	0.2034	−0.1667	0.078*	
C18	−0.2160 (3)	0.15871 (14)	−0.1662 (3)	0.0650 (6)	
H18A	−0.2133	0.1642	−0.2710	0.078*	
C19	−0.1107 (2)	0.12777 (14)	−0.0752 (3)	0.0592 (6)	
H19A	−0.0368	0.1133	−0.1185	0.071*	

### Atomic displacement parameters (Å^2^)

**Table:**

	*U*^11^	*U*^22^	*U*^33^	*U*^12^	*U*^13^	*U*^23^
Cl1	0.1158 (6)	0.0755 (5)	0.0653 (4)	−0.0226 (4)	−0.0141 (4)	0.0041 (3)
N1	0.0524 (11)	0.0529 (11)	0.0630 (12)	0.0028 (9)	0.0051 (9)	−0.0015 (10)
N2	0.0588 (12)	0.0757 (14)	0.0600 (12)	0.0135 (10)	0.0066 (10)	−0.0130 (10)
C1	0.0701 (15)	0.0421 (12)	0.0673 (16)	0.0003 (11)	0.0133 (12)	0.0000 (11)
C2	0.0662 (15)	0.0529 (14)	0.0643 (15)	0.0013 (12)	0.0057 (12)	0.0133 (12)
C3	0.0604 (14)	0.0540 (14)	0.0555 (14)	−0.0116 (11)	0.0057 (11)	0.0039 (11)
C4	0.0668 (15)	0.0431 (13)	0.0659 (15)	−0.0022 (11)	0.0121 (12)	−0.0037 (11)
C5	0.0508 (13)	0.0477 (13)	0.0633 (14)	0.0054 (10)	0.0072 (11)	0.0043 (11)
C6	0.0457 (12)	0.0469 (13)	0.0594 (14)	−0.0024 (10)	0.0116 (10)	0.0018 (11)
C7	0.0589 (14)	0.0455 (13)	0.0628 (15)	−0.0044 (11)	0.0092 (11)	−0.0050 (11)
C8	0.0451 (12)	0.0494 (13)	0.0573 (14)	−0.0045 (10)	0.0066 (10)	−0.0005 (10)
C9	0.0581 (14)	0.0464 (13)	0.0653 (15)	0.0028 (11)	0.0083 (11)	−0.0064 (11)
C10	0.0578 (14)	0.0478 (13)	0.0645 (15)	0.0046 (11)	0.0005 (11)	0.0023 (11)
C11	0.0426 (12)	0.0540 (14)	0.0583 (14)	−0.0037 (10)	0.0091 (10)	−0.0028 (11)
C12	0.0481 (13)	0.0568 (14)	0.0677 (15)	0.0041 (11)	0.0082 (11)	−0.0126 (12)
C13	0.0460 (12)	0.0538 (14)	0.0681 (15)	0.0056 (11)	0.0056 (11)	−0.0019 (12)
C14	0.0481 (12)	0.0453 (12)	0.0562 (13)	−0.0049 (10)	0.0052 (10)	−0.0059 (10)
C15	0.0550 (13)	0.0522 (13)	0.0535 (13)	−0.0032 (11)	0.0119 (10)	−0.0031 (10)
C16	0.0528 (13)	0.0527 (14)	0.0691 (16)	0.0027 (11)	0.0087 (11)	−0.0079 (12)
C17	0.0684 (16)	0.0557 (14)	0.0676 (16)	0.0073 (12)	−0.0033 (13)	−0.0016 (12)
C18	0.0788 (17)	0.0640 (16)	0.0519 (14)	−0.0021 (13)	0.0072 (12)	−0.0003 (12)
C19	0.0574 (14)	0.0626 (15)	0.0603 (15)	−0.0054 (12)	0.0175 (11)	−0.0071 (12)

### Geometric parameters (Å, °)

**Table:**

Cl1—C3	1.736 (2)	C9—C10	1.381 (3)
N1—C7	1.260 (3)	C9—H9A	0.9300
N1—C8	1.417 (3)	C10—C11	1.385 (3)
N2—C11	1.399 (3)	C10—H10A	0.9300
N2—C14	1.402 (3)	C11—C12	1.388 (3)
N2—H2A	0.8600	C12—C13	1.367 (3)
C1—C2	1.377 (3)	C12—H12A	0.9300
C1—C6	1.384 (3)	C13—H13A	0.9300
C1—H1B	0.9300	C14—C15	1.385 (3)
C2—C3	1.373 (3)	C14—C19	1.389 (3)
C2—H2B	0.9300	C15—C16	1.376 (3)
C3—C4	1.377 (3)	C15—H15A	0.9300
C4—C5	1.376 (3)	C16—C17	1.373 (3)
C4—H4A	0.9300	C16—H16A	0.9300
C5—C6	1.391 (3)	C17—C18	1.368 (3)
C5—H5A	0.9300	C17—H17A	0.9300
C6—C7	1.463 (3)	C18—C19	1.370 (3)
C7—H7A	0.9300	C18—H18A	0.9300
C8—C9	1.382 (3)	C19—H19A	0.9300
C8—C13	1.397 (3)		
			
C7—N1—C8	121.7 (2)	C9—C10—C11	120.2 (2)
C11—N2—C14	128.39 (19)	C9—C10—H10A	119.9
C11—N2—H2A	115.8	C11—C10—H10A	119.9
C14—N2—H2A	115.8	C10—C11—C12	118.1 (2)
C2—C1—C6	121.4 (2)	C10—C11—N2	123.6 (2)
C2—C1—H1B	119.3	C12—C11—N2	118.2 (2)
C6—C1—H1B	119.3	C13—C12—C11	121.6 (2)
C3—C2—C1	119.0 (2)	C13—C12—H12A	119.2
C3—C2—H2B	120.5	C11—C12—H12A	119.2
C1—C2—H2B	120.5	C12—C13—C8	120.7 (2)
C2—C3—C4	121.2 (2)	C12—C13—H13A	119.6
C2—C3—Cl1	119.14 (19)	C8—C13—H13A	119.6
C4—C3—Cl1	119.66 (19)	C15—C14—C19	118.6 (2)
C5—C4—C3	119.2 (2)	C15—C14—N2	122.7 (2)
C5—C4—H4A	120.4	C19—C14—N2	118.7 (2)
C3—C4—H4A	120.4	C16—C15—C14	120.0 (2)
C4—C5—C6	121.0 (2)	C16—C15—H15A	120.0
C4—C5—H5A	119.5	C14—C15—H15A	120.0
C6—C5—H5A	119.5	C17—C16—C15	121.0 (2)
C1—C6—C5	118.2 (2)	C17—C16—H16A	119.5
C1—C6—C7	120.1 (2)	C15—C16—H16A	119.5
C5—C6—C7	121.7 (2)	C18—C17—C16	119.1 (2)
N1—C7—C6	122.8 (2)	C18—C17—H17A	120.4
N1—C7—H7A	118.6	C16—C17—H17A	120.4
C6—C7—H7A	118.6	C17—C18—C19	120.8 (2)
C9—C8—C13	117.5 (2)	C17—C18—H18A	119.6
C9—C8—N1	116.5 (2)	C19—C18—H18A	119.6
C13—C8—N1	126.0 (2)	C18—C19—C14	120.5 (2)
C10—C9—C8	121.8 (2)	C18—C19—H19A	119.8
C10—C9—H9A	119.1	C14—C19—H19A	119.8
C8—C9—H9A	119.1		
			
C6—C1—C2—C3	1.1 (4)	C9—C10—C11—N2	−177.3 (2)
C1—C2—C3—C4	0.7 (4)	C14—N2—C11—C10	−18.4 (4)
C1—C2—C3—Cl1	−179.97 (17)	C14—N2—C11—C12	164.8 (2)
C2—C3—C4—C5	−1.4 (3)	C10—C11—C12—C13	0.1 (3)
Cl1—C3—C4—C5	179.28 (17)	N2—C11—C12—C13	177.0 (2)
C3—C4—C5—C6	0.3 (3)	C11—C12—C13—C8	0.5 (3)
C2—C1—C6—C5	−2.1 (3)	C9—C8—C13—C12	−0.5 (3)
C2—C1—C6—C7	178.2 (2)	N1—C8—C13—C12	−179.1 (2)
C4—C5—C6—C1	1.4 (3)	C11—N2—C14—C15	−32.5 (3)
C4—C5—C6—C7	−178.9 (2)	C11—N2—C14—C19	150.9 (2)
C8—N1—C7—C6	179.28 (18)	C19—C14—C15—C16	−0.9 (3)
C1—C6—C7—N1	−179.3 (2)	N2—C14—C15—C16	−177.6 (2)
C5—C6—C7—N1	1.0 (3)	C14—C15—C16—C17	0.8 (3)
C7—N1—C8—C9	167.8 (2)	C15—C16—C17—C18	0.2 (4)
C7—N1—C8—C13	−13.6 (3)	C16—C17—C18—C19	−1.2 (4)
C13—C8—C9—C10	0.1 (3)	C17—C18—C19—C14	1.0 (4)
N1—C8—C9—C10	178.8 (2)	C15—C14—C19—C18	0.0 (3)
C8—C9—C10—C11	0.5 (3)	N2—C14—C19—C18	176.8 (2)
C9—C10—C11—C12	−0.6 (3)		

### Hydrogen-bond geometry (Å, °)

**Table:**

Cg1 and Cg3 are the centroids of the C1–C6 and C14–C19 rings, respectively.

**Table:**

*D*—H···*A*	*D*—H	H···*A*	*D*···*A*	*D*—H···*A*
C1—H1B···*Cg*3^i^	0.93	2.95	3.661 (2)	135
C16—H16A···*Cg*1^ii^	0.93	2.90	3.624 (2)	136

Symmetry codes: (i) −*x*, −*y*, −*z*+1; (ii) *x*−1, *y*, *z*−1.
